# Anti-malarial landscape in Myanmar: results from a nationally representative survey among community health workers and the private sector outlets in 2015/2016

**DOI:** 10.1186/s12936-017-1761-8

**Published:** 2017-04-25

**Authors:** ACTwatch Group, Louis Akulayi, Angela Alum, Andrew Andrada, Julie Archer, Ekundayo D. Arogundade, Erick Auko, Abdul R. Badru, Katie Bates, Paul Bouanchaud, Meghan Bruce, Peter Buyungo, Angela Camilleri, Emily Carter, Steven Chapman, Nikki Charman, Desmond Chavasse, Robyn Cyr, Kevin Duff, Gylsain Guedegbe, Keith Esch, Illah Evance, Anna Fulton, Hellen Gataaka, Tarryn Haslam, Emily Harris, Christine Hong, Catharine Hurley, Whitney Isenhower, Enid Kaabunga, Baraka D Kaaya, Esther Kabui, Beth Kangwana, Lason Kapata, Henry Kaula, Gloria Kigo, Irene Kyomuhangi, Aliza Lailari, Sandra LeFevre, Megan Littrell, Greta Martin, Daniel Michael, Erik Monroe, Godefroid Mpanya, Felton Mpasela, Felix Mulama, Anne Musuva, Julius Ngigi, Edward Ngoma, Marjorie Norman, Bernard Nyauchi, Kathryn A. O’Connell, Carolyne Ochieng, Edna Ogada, Linda Ongwenyi, Ricki Orford, Saysana Phanalasy, Stephen Poyer, Justin Rahariniaina, Jacky Raharinjatovo, Lanto Razafindralambo, Solofo Razakamiadana, Christina Riley, John Rodgers, Andria Rusk, Tanya Sensalire, Simon Rusk, Julianna Smith, Phok Sochea, Tsione Solomon, Raymond Sudoi, Martine Esther Tassiba, Katherine Thanel, Rachel Thompson, Mitsuru Toda, Chinazo Toda, Cynthia Ujuju, Marie-Alix Valensi, Vamsi Vasireddy, Cynthia Whitman, Cyprien Zinsou, Si Thu Thein, Hnin Su Su Khin, Aung Thi

**Affiliations:** 1 Population Services International, 1120 19th St NW Suite 600, Washington, DC 20036 USA; 2 Population Services International Myanmar, No. 16, West Shwe Gone Dine 4th Street, Yangon, Myanmar; 3 National Malaria Control Programme, Department of Public Health, Ministry of Health, Naypyidaw, Myanmar

**Keywords:** Anti-malarial, Oral artemisinin monotherapy, Artemisinin combination therapy, Chloroquine, Malaria testing

## Abstract

**Background:**

In 2015/2016, an ACTwatch outlet survey was implemented to assess the anti-malarial and malaria testing landscape in Myanmar across four domains (Eastern, Central, Coastal, Western regions). Indicators provide an important benchmark to guide Myanmar’s new National Strategic Plan to eliminate malaria by 2030.

**Methods:**

This was a cross-sectional survey, which employed stratified cluster-random sampling across four regions in Myanmar. A census of community health workers (CHWs) and private outlets with potential to distribute malaria testing and/or treatment was conducted. An audit was completed for all anti-malarials, malaria rapid diagnostic tests.

**Results:**

A total of 28,664 outlets were approached and 4416 met the screening criteria. The anti-malarial market composition comprised CHWs (41.5%), general retailers (27.9%), itinerant drug vendors (11.8%), pharmacies (10.9%), and private for-profit facilities (7.9%). Availability of different anti-malarials and diagnostic testing among anti-malarial-stocking CHWs was as follows: artemisinin-based combination therapy (ACT) (81.3%), chloroquine (67.0%), confirmatory malaria test (77.7%). Less than half of the anti-malarial-stocking private sector had first-line treatment in stock: ACT (41.7%) chloroquine (41.8%), and malaria diagnostic testing was rare (15.4%). Oral artemisinin monotherapy (AMT) was available in 27.7% of private sector outlets (Western, 54.1%; Central, 31.4%; Eastern; 25.0%, Coastal; 15.4%). The private-sector anti-malarial market share comprised ACT (44.0%), chloroquine (26.6%), and oral AMT (19.6%). Among CHW the market share was ACT (71.6%), chloroquine (22.3%); oral AMT (3.8%). More than half of CHWs could correctly state the national first-line treatment for uncomplicated falciparum and vivax malaria (59.2 and 56.9%, respectively) compared to the private sector (15.8 and 13.2%, respectively). Indicators on support and engagement were as follows for CHWs: reportedly received training on malaria diagnosis (60.7%) or national malaria treatment guidelines (59.6%), received a supervisory or regulatory visit within 12 months (39.1%), kept records on number of patients tested or treated for malaria (77.3%). These indicators were less than 20% across the private sector.

**Conclusion:**

CHWs have a strong foundation for achieving malaria goals and their scale-up is merited, however gaps in malaria commodities and supplies must be addressed. Intensified private sector strategies are urgently needed and must be scaled up to improve access and coverage of first-line treatments and malaria diagnosis, and remove oral AMT from the market place. Future policies and interventions on malaria control and elimination in Myanmar should take these findings into consideration across all phases of implementation.

**Electronic supplementary material:**

The online version of this article (doi:10.1186/s12936-017-1761-8) contains supplementary material, which is available to authorized users.

## Background

Myanmar bears the highest malaria burden in the Greater Mekong Sub-region (GMS), accounting for around 70% of reported cases in the region. The incidence of reported malaria has dropped by about 49% since 2012 (from 8.09 in 2012 to 4.16 in 2015 per 1000 population) [1]. Approximately 16% of Myanmar’s population of 57 million live in areas of high transmission and another 44% live in areas of low transmission. *Plasmodium falciparum* makes up 75% of the parasite species while *Plasmodium vivax* comprises the other 25% [2].

In 2008, artemisinin-based combination therapy (ACT) (artemether–lumefantrine [AL], dihydroartemisinin–piperaquine [DHA-PP] or artesunate-mefloquine [ASMQ]) was introduced as the first-line treatment for uncomplicated falciparum malaria and chloroquine has been the first-line treatment for vivax malaria [2]. The 2012 Myanmar National Treatment Guidelines specify that a single dose of primaquine should be administered following confirmed cases of falciparum malaria and a 14-day dose for radical cure of vivax malaria. Policies have been implemented for the use of primaquine at varying levels of the health system, allowing the Government to limit use of primaquine to facilities that are equipped to either test and/or monitor for signs of glucose-6 phosphate dehydrogenase (G6PD) deficiency. However, as G6PD testing currently is seldom available in the field, implementation of this recommendation is limited [3].

To date, several strategies have been in place to ensure the appropriate diagnosis and treatment of malaria in Myanmar. One of the key interventions in Myanmar, through the National Malaria Control Programme (NMCP) as well as several non-governmental organizations (NGOs), has been the training and deployment of community health workers (CHWs) who complement the care provided by public healthcare workers in rural locations, which bear the greatest burden of disease [1, 2]. Since 2008, the primary role of these CHWs has been to provide access to confirmatory testing and first-line treatment for patients who present with symptoms of vivax or falciparum malaria. CHWs are part of public sector health services, but the providers themselves are volunteers who depend on the support of a NGO or the NMCP [4].

In the private sector, where up to 70% of Myanmar’s population receive treatment [5, 6], several initiatives have also been in place over recent years to strengthen malaria case management. In 2010, the Government of Myanmar developed a set of comprehensive interventions outlined in the “Myanmar Artemisinin Resistance Containment (MARC)” framework [7]. This included several activities to strengthen malaria case management services, including the aforementioned scale-up of community health workers. As part of the MARC framework, in 2012, Population Services International (PSI), a US-based NGO, began implementation of the artemisinin monotherapy replacement (AMTR) project. The aim of the AMTR project was to distribute highly subsidized, first-line ACT into the private sector and phase out oral artemisinin monotherapy (AMT). Prior to the intervention, it was estimated that up to 2.4 million packages of oral AMT were being distributed annually in Myanmar [8]. The AMTR project aimed to remove oral AMT from the market through price competition, intensive provider behaviour change communication and other demand-creation activities [5]. This was complemented with a ban in 2012 by the Government of Myanmar on oral AMT in an attempt to curb the widespread availability and use of this medicine [2]. While subsidized distribution of ACT occurred throughout the country, intensive provider behaviour change activities were limited to the eastern region of the country. These concerted efforts resulted in an increase in availability and distribution of ACT and a reduction in oral AMT in the eastern regions of Myanmar since 2012, though oral AMT still has a presence on the market [6]. Furthermore, in 2015, the AMTR project focused on increasing and scaling-up access to malaria confirmatory testing across certain parts of the country and 60,000 free rapid diagnostic tests (RDTs) were distributed in the private sector.

Despite several public and private-sector initiatives to better manage patients through appropriate treatment and malaria testing, the spread of artemisinin resistance in Myanmar is now apparent. While artemisinin resistance was thought to only exist on the Thailand–Myanmar border, with many of the aforementioned strategies over the past half-decade having focused heavily on this area, resistance has now been detected in areas close to the border with India [9]. This is of grave concern given Myanmar is noted as the anti-malarial resistance gateway to the Indian sub-continent and beyond, and thus is critical to global malaria control and elimination. Detection of artemisinin resistance, and the country’s commitment to eliminate malaria by 2030, has prompted an emergency re-assessment of malaria control and elimination strategies [4].

Key strategies to address malaria control and elimination efforts in Myanmar are outlined in the National Strategic Plan for Intensifying Malaria Control and Accelerating Progress towards Malaria Elimination (2016–2020) [1]. In the public sector, this includes scale-up of the CHW programme to improve coverage and access to appropriate malaria testing and treatment. The private sector will be increasingly regulated and licensed, with only ‘selected’ private-sector providers allowed to test and treat patients. Selected outlets include pharmacies, private companies and outlets, who will be trained, supervised, and provided with malaria commodities, and required to report on caseload data. In addition, the National Strategic Plan specifies that non-licensed drug vendors, except in special circumstances, will be prohibited from treating malaria and selling anti-malarial medicines. Several strategies will be undertaken to regulate non-licensed drug vendors, including enforcement through judiciary officers. Myanmar will also tighten the ban on oral AMT and implement police enforcement to stop the sale and distribution of oral AMT.

Timely and relevant anti-malarial market evidence will be useful to help provide a benchmark for Myanmar’s National Strategic Plan, to help accelerate progress towards elimination goals in the country and to prioritize strategic areas. Previous studies on the anti-malarial market and malaria diagnostics have been limited to the eastern part of the country [6] and, therefore, the performance CHWs and private-sector healthcare providers for malaria case management services across the country is largely unknown. Furthermore, the performance of the private sector across different geographical regions is likely to vary given the lack of uniform strategies to improve malaria case management, with most activities happening in the eastern part of the country.

The objective of this paper is to provide evidence to inform malaria elimination strategy and policy in Myanmar. The paper describes the market for malaria medicines and diagnostics among CHW and across the private sector. The potential of CHWs and the private sector in malaria control and elimination efforts is discussed.

## Methods

This study was a cross-sectional survey, which employed stratified cluster-random sampling across four regions (strata) in Myanmar. The study population consisted of a census of all anti-malarial stocking outlets in the selected clusters. The data collection lasted over five months, from late August 2015 to early January 2016.

ACTwatch project developed the methodology for this study [10, 11], and the same methodology was employed for three other studies conducted in the GMS in 2015/2016 [12]. The ACTwatch project is a multi-country research project, whose goal is to provide high-quality evidence on anti-malarial markets all over the world. Since its inception, the project has developed, applied and documented several standardized tools and approaches.

### Study population

**Fig. 1:**
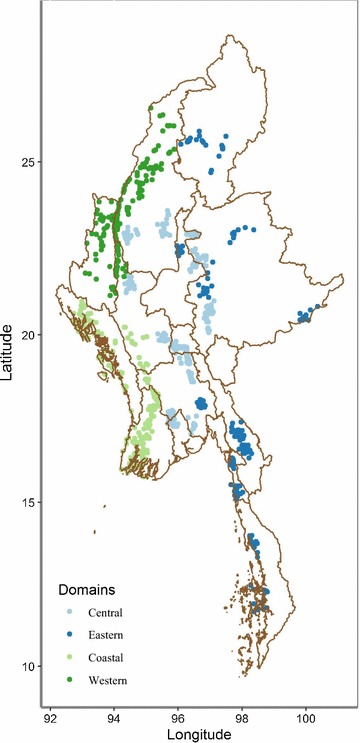
Map of selected clusters

The study used explicit stratification to provide estimates within four study regions: (1) Eastern areas were located primarily along the eastern border with Thailand and Yunan Province in China, which were part of the AMTR intervention programme activities and were expected to have different outcomes compared to other regions; (2) Central included areas of central Myanmar that were adjacent to the AMTR project area in eastern Myanmar but were not part of it, and were expected to have similar background characteristics to the Eastern region (in previous outlet survey studies, this region was typically considered a comparison region [6]); (3) Western included areas within Chin State, Sagaing, and Magway Regions which formed immediate or proximate borders with India; and, (4) Coastal, within Rakhine State, Magway, Bago and Ayeyarwaddy Regions which formed the border with Bangladesh and were part of the coastal area (Fig. 1).

### Eligibility criteria

All outlets with the potential to sell or distribute anti-malarial medicines were screened for eligibility. These included CHWs, private for-profit facilities, pharmacies, general retailers, and itinerant drug vendors (Table 1). All outlets, except government health facilities, were eligible for interview and an anti-malarial or RDT audit if they met at least one of three study criteria: (1) had one or more anti-malarial medicines in stock on the day of the survey; (2) had one or more anti-malarials reportedly in stock within the three months preceding the survey; and/or, (3) provided malaria blood testing, either microscopy or RDT. Public health facilities were excluded from the study because permission was not received to audit these facilities.

**Table 1:** Outlet types

Community health workers (CHWs)	Village-based volunteers who provide free or highly subsidized health services in remote rural areas. They are typically linked with government or non-government not-for-profit organizations, and usually receive training, support and supplies
Private sector	
Private-for-profit health facilities	General practitioners who operate within privately owned facilities that are licensed by Myanmar’s Ministry of Health. In some cases, the providers work for Ministry of Health as well and are running these clinics during their free time
Pharmacies	Pharmacies and drug stores, usually licensed by the Ministry of Health. They are usually small, privately owned, and stock various medicines including prescription-based ones
General retailers	Small grocery stores and village shops that sell fast-moving consumer goods, food and provisions. They often stock over-the-counter medicines including anti-malarial drugs but typically are not recognized as drug stores, nor hold licenses
Itinerant drug vendors	Informal healthcare providers who are mobile and typically operate in rural areas and cover more than one village. Some are retired healthcare providers from various government ministries but are no longer registered. They are typically not linked with regulatory authorities

In this study, private-for-profit health facilities, pharmacies, general retailers, and itinerant drug vendors comprise the ‘private sector’. CHWs are described separately as a different, public not-for profit channel given their mode of operation was different.

### Sample size

The study was designed to generate estimates for key market indicators within each region. Minimum sample size requirements were calculated to estimate, with ±10% precision, the following indicators: (1) the proportion of private-sector outlets with ACT availability, among outlets with anti-malarial(s) in stock on the day of the survey; and, (2) the proportion of private-sector outlets with oral AMT in stock, among outlets with anti-malarial(s) in stock on the day of the survey. The minimum number of outlets that needed to be screened was determined from the required number of anti-malarial stocking outlets, and the proportion of screened outlets that had anti-malarial(s) from previous studies [13]. That number was then divided by an estimated average number of private-sector outlets per cluster to attain the minimal number of clusters required for the study. In total, 836 clusters were selected across the four regions.

### Sampling approach

Clusters were selected using probability proportionate to size (PPS). A cluster was defined as a ‘ward’ in urban areas (towns and cities), and as a ‘village tract’ (a cluster of several villages) in rural areas. On average, 3000–5000 people resided in each cluster, but there were geographical differences.

The sampling frames were constructed using population sizes of clusters because the estimated number of outlets for each cluster did not exist. The population figures were used as a proxy measure with the assumption that the number of outlets within a given cluster were correlated with its population size. To manage the size of the survey and maintain quality, the survey was implemented in two phases, with the Eastern and Central regions in phase 1, and Western and Coastal regions in phase 2. Each phase had a slightly different sampling approach due to limited availability of population data for the sampling frame.

Phase 1 data collection used two-stage sampling, where larger clusters (townships) formed the first-stage sampling frame. From that, 28 townships were randomly selected using PPS. In the second stage, all wards and village tracts within the selected townships were listed, and systematic random sampling was used to select a fixed number of clusters from each township, resulting in the final sample of 448 clusters.

Phase 2 data collection used one-stage sampling as the actual population numbers of wards and village tracts had become available at that time from the 2014 Population and Housing Census. Consequently, the sampling frame consisted of all clusters (wards and village tracts) from each region, and a total of 360 clusters were randomly selected using PPS.

### Data collection

Two separate interviewer-training sessions were given, which spanned a total of eight days. The training focused on identification of outlets and anti-malarial medicines, informed consent procedures, and step-by-step walk-through of a full questionnaire.

Within each selected cluster, a census of all outlets with the potential to sell or distribute anti-malarials and/or provide malaria blood testing was conducted. The census involved systematically looking for outlets in each cluster, and using screening questions to identify outlets for inclusion in the study. Provider interviews and anti-malarial audits were conducted in all eligible outlets, after informed consent procedures.

For each eligible outlet, interviewers conducted an exhaustive audit of all anti-malarials and RDTs in stock at the time of the survey. For each and every anti-malarial medicine, the audit included formulation, brand name, active ingredients and strengths, manufacturer, and country of manufacture. The audit also collected information on unit costs of anti-malarials, and amount distributed to individual patients within the previous seven days. Basic outlet and provider characteristics, including availability of malaria microscopy, were collected. Questions related to private sector support and engagement were also administered to providers. Paper-based questionnaires and field monitoring sheets were used to record information.

### Data entry, processing and analysis

Double data entry and verification was performed using customised CSPro data entry forms. All data cleaning and analysis were completed using Stata 13.1 (©StataCorp, College Station, TX, USA). 2014 UNFPA census data was used to calculate sampling weights, applied at the township level to account for variations in probability of selection. Stata survey settings were used to reflect the study design and sampling approach, to compute estimates, including those at region-level. Standard error estimation, including application of a finite population correction, accounted for clustering at the ward/village track level. Weighting and finite population correction yielded confidence intervals (CI) used for comparison of proportions.

Standard indicators were constructed according to ACTwatch definitions [10, 11, 14]. All audited anti-malarial medicines were verified and classified using information on drug formulation, contents and strengths with supporting information, including brand or generic name and manufacturer. Anti-malarials were classified as ACT, non-artemisinin therapy, and oral or non-oral AMT. A generic classification of ACT was used as national policy for uncomplicated falciparum malaria was AL, PHA-PPQ or ASMQ. Availability of any anti-malarial was defined in this study as the proportion of outlets stocking at least one anti-malarial among all screened outlets. Other anti-malarial and RDT availability categories were calculated but restricted to those outlets where at least one anti-malarial was audited. For example, ACT availability (the proportion of ACT-stocking outlets) was measured as the number of ACT-stocking outlets in the numerator and the number of anti-malarial stocking outlets in the denominator.

Market share was defined as the relative distribution of the anti-malarials sold to individual consumers in the week preceding the survey. In order to allow for meaningful market share comparisons between products, information about anti-malarial distribution was standardized to the adult equivalent treatment dose (AETD). AETD is the amount of active ingredient necessary to treat a 60-kg adult according to World Health Organization (WHO) treatment guidelines [14]. Volumes distributed were calculated by converting provider reports on the number of anti-malarials sold in the week prior to the survey into AETDs. Volumes were the number of AETDs sold or distributed by a provider in the seven days prior to the survey. All dosage forms were considered in measuring volumes so as to provide a complete assessment of anti-malarial market share. Primaquine distribution was not included in calculations of total and relative volumes distributed. This is because primaquine is to be used only in combination with either an ACT for falciparum malaria, or with chloroquine for all other infections. Therefore, similar to the treatment of partner drugs within an ACT, we only consider volumes distributed for primaquine’s partner drugs (ACT or chloroquine).

Provider knowledge was assessed by administering knowledge questions to the senior-most provider at all anti-malarial-stocking outlets. Providers were asked to state the national first-line treatment and dosing regimen for uncomplicated falciparum/vivax malaria for a 60-kg adult. Providers citing any first-line ACT as the first-line treatment for falciparum malaria, or chloroquine for vivax malaria, were classified as having correct knowledge.

### Ethical considerations

The study was approved by PSI Research Ethical Board registered under the Office of Human Research Protections (OHRP FWA00009154, IRB#00006961). All interviews and product audits were conducted only after receiving verbal informed consent from the participating providers. Confidentiality and anonymity was maintained through all phases of the study, and all standard ethical guidelines were followed.

## Results

A total of 28,664 outlets that had potential to sell/distribute anti-malarial medicines were approached to participate in the survey across the four regions (Table 2). Of these, 28,267 outlets were screened for stocking anti-malarials or malaria diagnostic testing (309 outlets were closed at the time of visit or closed permanently, and 88 outlet providers refused). Of these, 4416 met the screening criteria and 4395 were interviewed. The number of interviewed outlets was highest in the more populous Eastern region (N = 1330), and lowest in Central region (N = 594). Among the interviewed outlets, 3859 were found to have at least one anti-malarial in stock at the time of the survey, 413 outlets had no anti-malarials in stock at the time but reported having stocked an anti-malarial in the past three months, and 123 had malaria diagnostic testing but no anti-malarials. Among outlets stocking anti-malarials or malaria tests on the day of survey, 8735 anti-malarial products and 1635 RDTs were audited.

**Table 2:** Total outlet survey sample

	Eastern	Central	Western	Coastal	Total
Outlets enumerated	8432	7481	5666	7085	28,664
Outlets screened	8271	7393	5598	7005	28,267
Outlets that met screening criteria	1563	710	1149	994	4416
Outlets interviewed	1554	702	1147	992	4395
Outlets that had any anti-malarial at the time of survey	1330	594	1065	870	3859

Among all screened outlets, anti-malarial availability was as follows: CHW, 45% (N = 2737); private for-profit facilities, 50.4%, (N = 610); pharmacies, 46.9% (N = 970); general retailers, 4.6% (N = 22,733); and itinerant drug vendors, 33.7% (N = 1217).

### Anti-malarial market composition

**Fig. 2:**
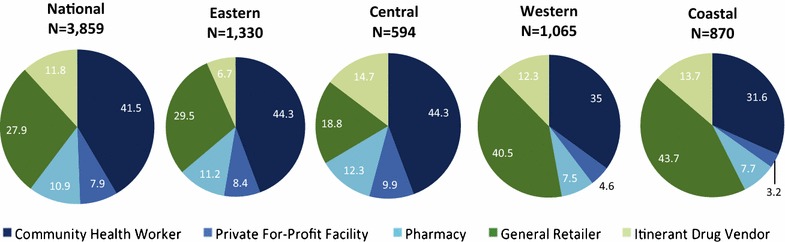
Anti-malarial market composition

Figure 2 shows the relative distribution of all outlets that had at least one anti-malarial in stock, by region and nationally. Estimates indicate that CHWs comprised 41.5% of the market composition, while other anti-malarial-stocking outlets were from the private sector (58.5%), including general retailers (27.9%), itinerant drug vendors (11.8%), pharmacies (10.9%), and private for-profit facilities (7.9%).

Observing regional differences, Eastern and Central illustrated a slightly higher market composition of CHWs (44.3 and 44.3 %, respectively) compared to Western and Coastal (35.0% and 31.6%, respectively). General retailers comprised the majority of the market composition in Western (40.5%) and Central (43.7%). Itinerant drug vendors comprised between 6.7 and 14.7% of the market composition across regions.

### Availability of anti-malarial medicines and diagnostics

Availability of anti-malarial medicines and malaria diagnostics among outlets stocking at least one anti-malarial is shown in Tables 3 and 4. Among anti-malarial-stocking CHWs, 83.1% stocked an ACT and 67.0% stocked chloroquine. Oral AMT was available in less than 5% of CHWs (2.9%). Around three out of four CHWs stocked a malaria blood test (RDT or microscopy) (77.7%). There were few regional differences between indicators among CHWs (Table 3).

**Table 3:** CHW availability of anti-malarial drugs and malaria diagnostics, among anti-malarial-stocking outlets, by region

	CHW Total(N = 1263) (95% CI)	Eastern (N = 545) (95% CI)	Central (N = 244) (95% CI)	Western (N = 242) (95% CI)	Coastal N = (232) (95% CI)
Availability of anti-malarials^a^					
Any ACT^b^	83.1 (80.1, 86.1)	87.8 (83.9, 90.9)	76.2 (69.8, 81.6)	83.3 (76.1, 88.6)	91.7 (86.2, 95.1)
Chloroquine	67.0 (63.5, 70.5)	67.6 (63.0, 71.8)	65.4 (58.8, 71.4)	76.0 (68.4, 82.3)	67.3 (59.0, 74.6)
Primaquine	62.6 (58.6, 66.5)	60.3 (55.3, 65.2)	58.1 (50.4, 65.3)	72.7 (64.2, 79.8)	74.7 (67.5, 80.8)
Oral AMT	2.9 (1.6, 4.2)	2.4 (1.2, 4.7)	4.0 (2.1, 7.5)	4.5 (2.6, 7.9)	0.6 (0.2, 2.2)
Non-oral AMT	8.9 (6.5, 11.3)	6.5 (4.1, 10.2)	12.3 (8.3, 17.8)	7.9 (5.1, 12.0)	5.0 (2.4, 10.2)

	N = 1382	N = 597	N = 277	N = 257	N = 251
Availability of blood testing^c^					
Any malaria blood testing	77.7 (74.4, 81.0)	79.3 (75.5, 82.7)	72.5 (65.6, 78.4)	83.6 (78.1, 87.9)	86.1 (79.7, 90.7)
Malaria microscopy	0.2 (0.0, 0.5)	0.1 (0.0, 0.7)	0.3 (0.0, 2.1)	1.1 (0.2, 5.9)	0.0
RDTs	77.7 (74.4, 81.0)	79.3 (75.5, 82.7)	72.5 (65.6, 78.4)	83.6 (78.1, 87.9)	86.1 (79.7, 90.7)

*AMT* artemisinin monotherapy

^a^Anti-malarial-stocking outlets have at least one anti-malarial in stock on the day of the survey, verified by presence of at least one anti-malarial recorded in the ant-imalarial audit sheet

^b^At the time of the 2015/2016 Myanmar ACTwatch outlet survey, AL, DHA-PP, and ASMQ were the first-line treatments for uncomplicated falciparum malaria. There was no ASMQ audited during the 2015/2016 survey

^c^Blood testing availability is reported among outlets that either had anti-malarials in stock on the day of the survey or reportedly stocked anti-malarials in the previous 3 months

**Table 4:** Private sector availability of anti-malarial drugs and malaria diagnostics, among anti-malarial-stocking outlets

	Private Sector Total N = 2596 (95% CI)	Eastern (N = 785) (95% CI)	Central (N = 350) (95% CI)	Western (N = 823) (95% CI)	Coastal N = (638) (95% CI)
Availability of anti-malarials^a^					
Any ACT^b^	41.7 (36.9, 46.6)	65.6 (59.5, 71.2)	36.7 (27.6, 46.8)	14.1 (11.2, 17.5)	19.0 (14.2, 25.0)
Chloroquine	41.8 (38.4, 45.3)	24 (20.1, 28.3)	43.5 (37.0, 50.2)	47.7 (41.6, 53.8)	68.6 (62.4, 74.2)
Primaquine	7.7 (4.9, 10.4)	7.5 (5.4, 10.4)	11.1 (6.4, 18.4)	2.9 (1.6, 5.2)	1.0 (0.5, 1.9)
Oral AMT	27.7 (23.8, 31.7)	25.0 (18.9, 32.4)	31.4 (24.8, 38.9)	54.1 (49.4, 58.7)	15.4 (11.7, 20.2)
Non-oral AMT	11 (8.6, 13.4)	11.7 (7.7, 17.3)	11.8 (8.6, 16.2)	18.3 (15.0, 22.2)	5.5 (3.3, 9.0)

	N = 2890	N = 904	N = 398	N = 877	N = 711
Availability of blood testing^c^					
Any malaria blood testing	15.4 (12.6, 18.1)	21.0 (17.2, 25.4)	14.8 (10.4, 20.5)	8.4 (6.0, 11.7)	8.7 (5.7, 13.0)
Malaria microscopy	0.6 (0.2, 0.9)	0.6 (0.3, 1.2)	0.7 (0.2, 2.0)	0.7 (0.3, 1.5)	0.2 (0.0, 0.8)
RDTs	14.9 (12.3, 17.6)	20.5 (16.7, 24.8)	14.2 (9.9, 19.8)	8.2 (5.8, 11.4)	8.7 (5.7, 13.0)

*AMT* artemisinin monotherapy

^a^Anti-malarial-stocking outlets have at least one anti-malarial in stock on the day of the survey, verified by presence of at least one anti-malarial recorded in the anti-malarial audit sheet

^b^At the time of the 2015/2016 Myanmar ACTwatch outlet survey, AL, DHA-PP, and ASMQ were the first-line treatments for uncomplicated falciparum malaria. There was no ASMQ audited during the 2015/2016 survey

^c^Blood testing availability is reported among outlets that either had anti-malarials in stock on the day of the survey or reportedly stocked anti-malarials in the previous 3 months

ACT was available in fewer than half of the anti-malarial-stocking private sector outlets (41.7%) (Table 4). ACT availability was highest in Eastern (65.6%), followed by Central (36.7%), Coastal (19.0%) and Western (14.1%) regions. Chloroquine was found in 41.8% of private-sector outlets. Chloroquine availability was highest in Coastal (68.6%), followed by Western (47.7%), Central (43.5%) and Eastern (24.0%). Availability of primaquine was rare (7.7%). Oral AMT availability was found in 27.7% of the private sector, and ranged from 54.1% of anti-malarial-stocking outlets in the Western region to 15.4% in the Coastal region. Availability of non-oral AMT was less than 20% across the private sector, and highest in the Western region (18.3%). Malaria blood testing was available in 15.4% of the anti-malarial-stocking private sector outlets: RDT (14.9%) rather than microscopy (0.6%). Malaria blood testing was highest in the Eastern region (20.5%) and lowest in the Western and Coastal regions (<10%).

### Anti-malarial market share

**Fig. 3:**
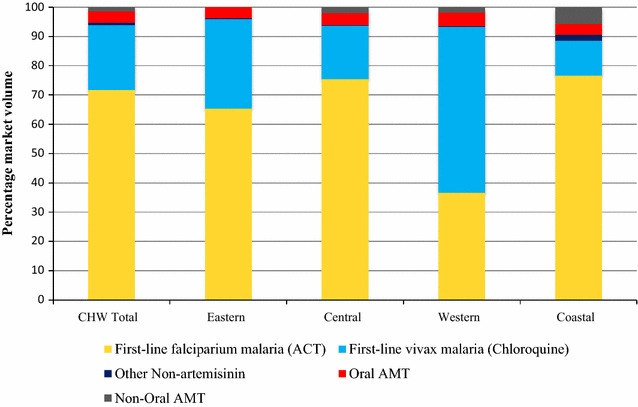
Anti-malarial market share: CHW

Figure 3 illustrates the market share of different categories of anti-malarial medicines sold or distributed within seven days prior to the survey among CHW. 71.6% of the market share comprised ACT, followed by chloroquine (22.3%). Distribution of oral AMT was rare, 3.7% of the market share. ACT market share was lowest in the Western region (36.6%) compared to other regions which had an ACT market share greater than 60%.

**Fig. 4:**
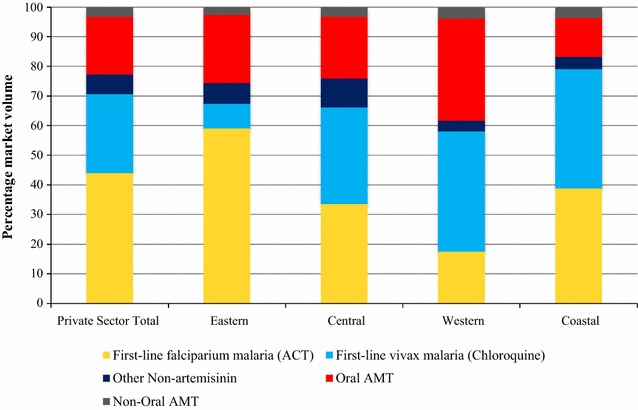
Anti-malarial market share: Private Sector

Figure 4 illustrates the market share of different categories of anti-malarial medicines sold or distributed within seven days prior to the survey in the private sector. The national private-sector anti-malarial market share comprised ACT (44.0%), chloroquine (26.6%), oral AMT (19.6%). ACT market share was highest in the Eastern region, accounting for 59.1% of the market share and lowest in Western region (17.6%). Chloroquine market share was lowest in the Eastern region (8.3%) but similar across other regions, ranging from 32.6 to 40.5%. Oral AMT was distributed across all regions, and highest in the Western region (34.5%) and lowest in the Coastal region (13.1%). Non-oral AMT market share was less than 10% across regions.

The relative private sector market share across outlet types is also presented, excluding contributions from CHW (Additional file 1). In the private sector, the majority of anti-malarials were distributed by pharmacies (39.1%). Private-for-profit facilities, general retailers and itinerant drug vendors accounted for around 60% of the total private sector market share (19.1, 21.8 and 20.3%, respectively).

### Providers’ knowledge

More than half of CHWs could correctly state the national first-line treatment for uncomplicated falciparum and vivax malaria (59.2 and 56.9%, respectively) (Table 5). Less than 20% of private-sector providers could correctly state the first-line treatment for uncomplicated falciparum and vivax malaria (15.8 and 13.2%, respectively) (Table 6). Region-specific differences within the private sector were also present, where less than 7% of providers in the Western and Coastal regions were able to correctly state the first-line treatment. There were few regional differences by CHW.

**Table 5:** CHW knowledge of the first-line treatment guidelines

	CHW Total N = 1382 (95% CI)	Eastern (N = 597) (95% CI)	Central (N = 277) (95% CI)	Western (N = 257) (95% CI)	Coastal N = (251) (95% CI)
Proportion of providers who:					
Correctly stated national first-line treatment for uncomplicated falciparum malaria	59.2 (55.1, 63.2)	60.7 (55.5, 65.7)	57.2 (49.8, 64.3)	65.7 (57.9, 72.7)	51.7 (43.1, 60.3)
Correctly stated national first-line treatment for uncomplicated vivax malaria	56.9 (52.9, 60.9)	60.5 (55.9, 64.1)	56.9 (49.3, 64.1)	64.9 (57.0, 73.2)	50.4 (42.3, 58.5)

**Table 6:** Private sector knowledge of the first-line treatment guidelines

	Private sector total N = 2596 (95% CI)	Eastern (N = 904) (95% CI)	Central (N = 398) (95% CI)	Western (N = 877) (95% CI)	Coastal N = (711) (95% CI)
Proportion of providers who:					
Correctly stated national first-line treatment for uncomplicated falciparum malaria	15.8 (12.6, 19.0)	19.8 (16.7–23.3)	18.3 (12.6–26.0)	5.0 (3.3–7.4)	5.5 (3.3–8.9)
Correctly stated national first-line treatment for uncomplicated vivax malaria	13.2 (10.5, 15.9)	14.7 (11.6, 18.4)	14.7 (10.1, 20.8)	4.3 (2.7, 6.6)	6.3 (4.2, 9.4)

### Supportive supervision and passive surveillance

The majority of CHW (60.7%) reportedly receiving training on malaria diagnosis, and 59.6% on national malaria treatment guidelines (Table 7). Almost 40% reportedly received a supervisory or regulatory visit within 12 months. Similarly, 77.3% reportedly kept records on number of patients tested or treated for malaria and 76.0% said they reported these numbers to government (51.8%) or NGOs (26.2%). There were few regional differences among CHW.

**Table 7:** CHW supervision, support and caseload reporting

	CHW total % (95% CI)	Eastern % (95% CI)	Central % (95% CI)	Western % (95% CI)	Coastal % (95% CI)
	N = 1461	N = 625	N = 301	N = 264	N = 271
Proportion of providers who:					
Trained on malaria diagnosis (RDT and/or microscopy)	60.7 (57.0, 64.4)	63.4 (58.7, 67.9)	56.5 (49.5, 63.2)	66.4 (58.0, 73.8)	64.5 (56.3, 71.8)

	N = 1461	N = 624	N = 301	N = 264	N = 272
Trained on national malaria treatment guidelines	59.6 (55.8, 63.4)	62.3 (57.3, 67.1)	57.3 (50.3, 64.1)	60.5 (51.4, 68.8)	60.1 (52.5, 67.3)

	N = 1458	N = 626	N = 300	N = 263	N = 269
Reported receiving a supervisory or regulatory visit within the past year	39.1 (35.8, 42.4)	59.5 (53.6, 65.0)	28.3 (23.4, 33.8)	38.6 (31.2, 46.5)	28.8 (22.4, 36.3)

	N = 1461	N = 625	N = 301	N = 264	N = 271
Kept records on number of patients tested/treated for malaria	77.3 (74.1, 80.6)	76.5 (71.9, 80.7)	74.8 (68.4, 80.2)	82.4 (76.5, 87.1)	83.7 (75.9, 89.3)

	N = 1459	N = 623	N = 301	N = 264	N = 271
Reported numbers of patients tested/treated for malaria to government or NGO	76.0 (72.6, 79.3)	75.6 (70.9, 79.7)	73.5 (67.0, 79.1)	81.4 (75.3, 86.2)	81.2 (72.9, 87.4)

	N = 1459	N = 623	N = 301	N = 264	N = 271
Reported numbers to government	51.8 (48.1, 55.5)	40.3 (35.5, 45.2)	57.7 (51.3, 63.9)	38.1 (30.9, 45.9)	62.5 (52.8, 71.2)

	N = 1459	N = 623	N = 301	N = 264	N = 271
Reported numbers of to a NGO	26.2 (22.6, 29.9)	36.4 (31.3, 41.8)	19.2 (13.9, 26.0)	43.2 (35.2, 51.7)	20.0 (12.6, 30.2)

Less than 10% of private-sector outlets reportedly received training within the past year, and supervisory/regulatory visits were reported by only one in four providers (19.9%) (Table 8). Only 12.2% kept patient records, and less than 10% reported to government (2.9%) or NGOs (6.6%). Private sector region-specific differences were observed, with highest numbers in Eastern region, with almost half of private-sector providers (47.8%) reporting supervisory/regulatory visits. However, all other indicators were typically less than 15%, with less than 6% outlets located in the Western and Central region reportedly receiving training on diagnosis, national treatment guidelines, receiving a supervisory visit, and reporting on caseload data.

**Table 8:** Private sector supervision, support and caseload reporting

	Private sector total % (95% CI)	Eastern % (95% CI)	Central % (95% CI)	Western % (95% CI)	Coastal % (95% CI)
	N = 2925	N = 923	N = 400	N = 882	N = 720
Proportion of providers who:					
Trained on malaria diagnosis (RDT and/or microscopy)	8.0 (5.9, 10.2)	12.0 (9.1, 15.7)	8.1 (4.8, 13.2)	4.8 (3.2, 7.1)	1.7 (0.9, 3.0)

	N = 2924	N = 922	N = 400	N = 882	N = 720
Trained on national malaria treatment guidelines	9.4 (6.9, 11.8)	12.9 (9.7, 16.8)	9.0 (5.3, 14.8)	5.2 (3.7, 7.4)	5.3 (2.8, 9.7)

	N = 2917	N = 921	N = 399	N = 879	N = 718
Reported receiving a supervisory or regulatory visit within the past year	19.9 (17.1, 22.7)	47.8 (41.2, 54.4)	8.7 (5.6, 13.4)	2.7 (1.4, 5.2)	2.0 (1.1, 3.8)

	N = 2920	N = 924	N = 397	N = 881	N = 718
Kept records on number of patients tested/treated for malaria	12.2 (9.3, 15.2)	16.3 (13.1, 20.1)	13.3 (8.5, 20.3)	6.4 (4.2, 9.6)	3.9 (1.8, 8.4)

	N = 2905	N = 921	N = 386	N = 880	N = 718
Reported numbers of patients tested/treated for malaria to government or NGO	9.4 (6.7, 12.1)	13.2 (10.5, 16.6)	9.7 (5.4, 16.6)	4.5 (2.6, 7.5)	3.1 (1.2, 7.8)

	N = 2905	N = 921	N = 396	N = 880	N = 718
Reported numbers to government	2.9 (1.7, 4.2)	2.7 (1.7, 4.3)	3.5 (1.8, 6.6)	2.4 (1.3, 4.6)	2.3 (0.6, 7.7)

	N = 2905	N = 921	N = 396	N = 880	N = 718
Reported numbers of to a NGO	6.6 (4.6, 8.6)	10.4 (7.9, 13.6)	6.6 (3.6, 11.8)	2.0 (0.8, 4.8)	0.8 (0.4, 1.8)

## Discussion

The 2015/2016 outlet survey presents, for the first time, national estimates of the anti-malarial market among CHWs and the private sector in Myanmar. Findings point to a strong foundation for malaria case management among CHWs but highlight key gaps in the private sector as well as notable regional differences. The results also point to the urgent need to remove oral AMT from the private sector marketplace.

### CHW readiness for appropriate malaria case management

Findings from the outlet survey illustrate the importance of CHWs, with up to 40% of the anti-malarial service delivery points comprising of these providers, though this contribution is likely to be lower if public health facilities were included in the sample. These providers were more prevalent in Eastern areas of the country, which reflect several MARC initiatives to scale-up these community-based providers.

Readiness for malaria case management implies having malaria commodities (first-line treatment for uncomplicated malaria and/or confirmatory testing) in stock at the time of survey. The findings point to strong readiness for appropriate malaria case management among CHWs who were found to be stocking anti-malarials. More than three-quarters had confirmatory testing available. Over 80% had first-line treatment for falciparum malaria in stock and more than half had the first-line treatment for vivax malaria. More than half of the CHW received training on national treatment guidelines and/or testing, and over three-quarters kept malaria case load data.

These findings suggest that there is merit in expanding and scaling-up CHWs further as a means to reach remote communities with malaria commodities, including expansion to the Western region where malaria endemicity is even higher than in other parts of the country. This recommendation is supported by several studies in Myanmar which have shown the CHW programme to be relatively inexpensive to implement [15], to have improved access to early and reliable diagnosis and treatment among marginalized groups [16] and to have improved malaria healthcare [17]. Key challenges to be addressed include ensuring a constant supply of first-line treatments, given over half of the CHWs were not stocking any anti-malarials on the day of survey or in the past 3 months. It is not clear from this study if this finding reflects long term stock-outs or rather inactive CHW. While over 40,000 CHW have been deployed over the years by the government and other partners in Myanmar, it is noteworthy that not all of these community based providers may be tasked with the provision of malaria commodities. Moreover, there is a high rate of attrition of CHWs and it was reported in 2015 that only 15,000 were currently active or functional as per the National Strategic Plan [1]. Several strategies may need to be considered to improve retention and motivation of CHWs, such as incentive schemes, training and supervision, and ensuring regular supply of commodities [18–21].

National guidelines stipulate that CHWs are authorized and recommended to provide a low dose of primaquine (0.25 mg) once weekly for eight weeks after chloroquine to prevent vivax malaria relapse, which has been found to be the front-line therapy for radical cure of *P. vivax* [22]. A single dose of primaquine following ACT for falciparum malaria is also recommended in the national treatment guidelines in order to substantially reduce transmission potential [23]. The outlet survey found that availability of primaquine among CHW however was moderate, around 60%. This may highlight challenges with procurement of the medicine to maintain constant supply, although availability of primaquine was much higher in Myanmar than in some neighbouring ACTwatch countries [24, 25]. This gap in CHW readiness to provide primaquine will be of importance to address given evidence that the addition of a single dose of primaquine could have a major effect on malaria transmission from falciparum malaria patients [26].

### Role of the private sector in appropriate malaria case management

Consistent with findings from other countries in the GMS, the private sector plays an important role in malaria case management [24, 25]. Results from this study show that the private-sector comprised over half of the anti-malarial service delivery points, and this was most notable in the Western and Coastal regions. Myanmar’s private sector was typically made up of pharmacies, general retailers and itinerant drug vendors, all of which were allowed to test and treat for malaria according to national policy at the time of the survey. Private sector market share data revealed that pharmacies distributed most of the private sector anti-malarials, however general retailers and itinerant drug vendors were also common sources, illustrating the need to reach these types of outlets as part of elimination strategies.

These findings have several implications for Myanmar’s malaria National Strategic Plan as it sets out to increase regulation of several private sector outlet types, and clamp down on outlets that are not licensed. Removing general retailers and itinerant drug vendors from the anti-malarial market, or making it illegal for them to sell anti-malarials or provide testing may result in a lack of access to malaria commodities. Several success stories have been demonstrated by the AMTR project which has specifically included these types of outlets as part of their strategy to promote ACT uptake through behaviour change communication and product promoter visits [5, 6]. While it may not be feasible to scale-up or replicate such an initiative to the entire country, ensuring that these anti-malarial-stocking providers have constant supply and access to malaria commodities may be an initial step to maintain existing levels of coverage and access to malaria treatment. Private sector training, capacity-building and demand generation will be important strategies to complement efforts to increase coverage of malaria commodities in the private sector [27].

### Readiness and performance of the private sector

The private sector was generally less well equipped to test and appropriately treat malaria infections compared with CHWs. Where anti-malarials were available in the private sector, fewer than half of anti-malarial-stocking outlets had first-line treatments available for falciparum or vivax malaria. There were, however, notable differences in availability of first-line treatments according to different geographical areas. Availability and market share of first-line treatment for falciparum malaria, ACT, was more common in the Eastern region than in the Western or Coastal regions. Over 60% of the anti-malarials distributed in Eastern Myanmar were ACT compared to 18% in the Western region. These findings are most likely attributable to several initiatives, including the AMTR project, which has included intensified activities across the Eastern part of Myanmar to increase demand and uptake of ACT, as previously mentioned.

Availability of malaria blood testing in the private sector was generally low, with 15% or less of outlets having RDT or microscopy available. These gaps in private sector readiness are a threat to appropriate management of suspected cases, given the real likelihood of presumptive anti-malarial treatment. In particular, while the market share data suggest that over 70% of anti-malarials distributed in the week prior to the survey were first-line treatments for falciparum or vivax malaria in the private sector, it is highly likely that most of these were given presumptively as providers did not have access to malaria tests. Without diagnostic blood tests, providers had no reliable way of differentiating the types of malaria infections. As the national malaria treatment guidelines are different for falciparum and vivax malaria, adhering to national treatment guidelines was inherently impossible for most private sector providers in the absence of confirmatory testing.

As the malaria National Strategic Plan stipulates universal coverage of malaria testing, several strategies are needed to scale-up coverage of diagnostics, including efforts that are already underway as part of the AMTR project to promote access of RDTs in the private sector. Strategies may include the provision of training and supervision to administer parasitological testing, as well as incentive models for providers, and maintaining constant supply of RDTs [28]. From the demand side this will require promoting RDTs as an important commodity for which patients are willing to pay for [29]. In fact, evidence suggests that the introduction of RDTs in Myanmar may be highly acceptable, even among the informal private sector, and could serve to promote provider empowerment and improve patient-provider relationships [30]. Other research has shown successful outcomes after introducing RDTs in the private sector [31, 32]. However, challenges with adhering to different treatment regimens for falciparum and vivax malaria based on RDT results, as well as a focus on what to do for a negative RDT result, are indicative of the need to promote training and supervision in light of any large-scale roll-out of RDTs in the private sector [33]. Lessons learnt from Cambodia’s experience of introducing RDTs in the private sector may be useful to review in light of any national scale-up of RDTs across Myanmar [28].

### Private sector availability and distribution of oral artemisinin monotherapy

Oral AMT poses a serious threat to the continued efficacy of artemisinins in Myanmar and across the GMS. Since 2008, the WHO has called for a ban on this monotherapy, and in 2012 Myanmar followed suit with other countries in the GMS, issuing a ban on the importation of oral AMT. However, data point to the widespread availability and distribution of this anti-malarial in the private sector, accounting for one in every four anti-malarials distributed. Results were most concerning across the Western region of the country, where one in three outlets were found to have oral AMT in stock, accounting for 34.5% of the market share. Oral AMT was also most commonly distributed among itinerant drug vendors, although other outlet types play an important role.

While several initiatives have been in place in Eastern Myanmar to remove this from the market, results point to the fact that oral AMT persists, with 25% of outlets stocking this in 2015/2016, an overall 17-point percentage increase from the previous sub-national survey implemented in project intervention areas of Eastern Myanmar [6]. The reasons for this increase are unclear. It is postulated that increases may be due to profit margins obtained from oral AMT versus highly subsided ACT, or a push by providers and manufactures to sell soon to be expired stock, or/and consumer demand for this medicine [34]. This may also reflect low levels of provider awareness about recommended first-line treatments and/or beliefs and preferences for non-first-line medicines [35–37]. Further research is being implemented to understand provider perceptions around oral AMT as a means to explain stocking and dispensing practices.

Perhaps of gravest concern is the possibility that actual market share of oral AMT was higher than estimated by the survey. The ACTwatch outlet survey analysis assumes a full course AETD to calculate the basic unit for market share. However, in reality the actual sale to patients may be less than a full course treatment. In Myanmar where ACT was commonly sold as full course treatments, the situation is rather different with oral AMT, which was typically dispensed as one or two tablets to a patient instead of the full AETD of 19.2 tablets with which oral AMT market share is calculated. Therefore, the proportion of patients that are treated with oral AMT relative to other types of anti-malarials is likely much higher than the market share estimated using AETDs distributed.

The results from this survey point to the fact that oral AMT remains a serious public health concern in Myanmar. Several reasons for the persistent availability and sale of this medicine have been postulated, including a relatively lenient ban which allows distributors to continue to import and sell this drug [6]. Action is urgently required to address this finding of grave public health significance.

### Provider knowledge

Provider knowledge was generally lower in the private sector compared to CHWs, with slightly fewer than half of these providers knowing the first-line treatment for either falciparum or vivax malaria. Other studies have shown that provider knowledge of medicines and doses, particularly in the private sector, is often poor [38, 39]. Indeed, in the private sector, knowledge was less than 20% and exceptionally low in the Western region, where less than 5% of providers could correctly state the first-line treatment for falciparum or vivax malaria. This speaks to the need to promote provider awareness of the first-line treatment regimens for falciparum or vivax malaria.

Increasing provider knowledge may be a first step towards ensuring the delivery of first-line treatments. That said, some studies have found no evidence of a relationship between providers’ knowledge and practice, and have suggested that provider preference is a stronger predictor of appropriate case management practices [40]. As such, simply increasing knowledge of the first-line treatment may have a limited effect, as supported by other studies [41–43]. This points to the importance of designing interventions that strive to change what providers think and believe to be appropriate, not only to enhance what they know. This could be complemented with widespread behaviour change communication, alerting communities to the first-line treatments, to the importance of receiving a confirmatory test prior to treatment, and dangers of oral AMT and sub-clinical dosing. Such multi-pronged strategies will be important in Myanmar to accelerate universal coverage of confirmatory testing and appropriate malaria treatment.

### Supervision and malaria caseload reporting

Overall private sector supervision, training on either national guidelines or diagnostic testing was low with fewer than one in five providers reporting these activities. The exception to this was in the Eastern region, where over half of the providers received a supervisory visit. This is most likely attributable to the AMTR supporting interventions, which includes routine visits from product promoters to pharmacies, general retailers and itinerant drug vendors.

These important benchmarks will be useful to guide future national strategy, which has proposed that the anti-malarial-stocking private sector should report on caseload data. Motivating these private-sector outlets will be key to ensure they report on testing and treatment outcomes. However, there are notable challenges with private sector caseload reporting, including a lack of provider incentives and operation of this sector outside the National Health Management Information Services (HMIS) [44]. Of promise is that several private sector initiatives are in place to do this, including the GMS Elimination of Malaria through Surveillance Programme (GEMS) which aims to actively increase malaria testing, treatment and reporting in the private sector through training, supervision and surveillance [45]. Caseload data from the private sector will be integrated with public sector data to provide national programmes with a more complete picture of malaria burden to respond to all detected cases.

### Study limitations

Some limitations are acknowledged. First, as the study excluded public health facilities due to operational constraints, the total anti-malarial market for the whole country could not be estimated. A follow-up survey that includes public health facilities would be useful to explore the readiness of the public sector and allow for the total anti-malarial market share to be calculated. Second, as the survey was cross-sectional it could not track the actual movement of drug stocks at the outlets. For this reason, all market share calculations were based on reported sales within one week and were subject to recall bias and volatility of the market. Lastly, the data collection period spanned more than four months from the end of August 2015 to early January 2016, and anti-malarial markets might have shifted during that time due to seasonal variations.

Regardless of the aforesaid limitations, the study was the first to produce national estimates of the anti-malarial market among CHWs and the private sector in Myanmar. As Myanmar has the highest burden of malaria cases in GMS and moves towards malaria elimination, the need for a comprehensive picture of entire malaria testing and treatment landscape was never more pressing.

## Conclusions

Results from this study suggest there are key gaps in private-sector readiness for appropriate malaria case management, and to some extent these gaps are also observed among CHWs. Availability of first-line treatments and malaria diagnostic tests was moderately high among the CHWs. These providers may be an important channel to reach remote rural communities, but it will be necessary to maintain constant supply of commodities to ensure universal coverage of confirmatory testing and national first-line treatment. The private sector remains responsible for most of malaria testing and treatment in Myanmar, and while most of the anti-malarials distributed were first-line treatments, availability of confirmatory testing was rare, meaning that most patients are being treated presumptively with either chloroquine or ACT. Of great urgency is the need to remove the widespread availability and distribution of oral AMT, which threatens global progress towards malaria control and case management. Poor private-sector knowledge, combined with lack of training or supervision, further compounds the situation. While several strategies have focused on strengthening the private sector in the eastern part of the country, and results from the private sector in this area are more promising, these strategies must be intensified and scaled up, using a multipronged approach to promote both provider and consumer behaviour change. Future policies and interventions on malaria control and elimination in Myanmar should take these factors into consideration across all phases of implementation.

## Additional file

**Additional file 1.** Anti-malarial market share across private sector outlet types.

## Abbreviations

AETD: adult equivalent treatment dose
ACT: artemisinin-based combination therapy
AL: artemether–lumefantrine
AMTR: artemisinin monotherapy replacement project
AMT: artemisinin monotherapy
ASMQ: artesunate–mefloquine
CHWs: community health workers
CI: confidence interval
DHA-PP: dihydroartemisinin-piperaquine
G6PD: glucose-6 phosphate dehydrogenase
GEMS: GMS Elimination of Malaria through Surveillance Programme
GMS: Greater Mekong Sub-region
HMIS: National Health Management Information Services
NMCP: National Malaria Control Programme
NGO: non-government organization
MARC: Myanmar artemisinin resistance containment
PSI: Population Services International
PPS: probability proportionate to size
RDTs: rapid diagnostic tests
WHO: World Health Organization
