# Supplementary material for: Anti-malarial landscape in Myanmar: results from a nationally representative survey among community health workers and the private sector outlets in 2015/2016
Source: Malar J. 2017 Apr 25;16:129. doi: 10.1186/s12936-017-1761-8 (PMC5404301; doi:10.1186/s12936-017-1761-8)
Supplement: Supplementary file 1 [file 12936_2017_1761_MOESM1_ESM.docx]

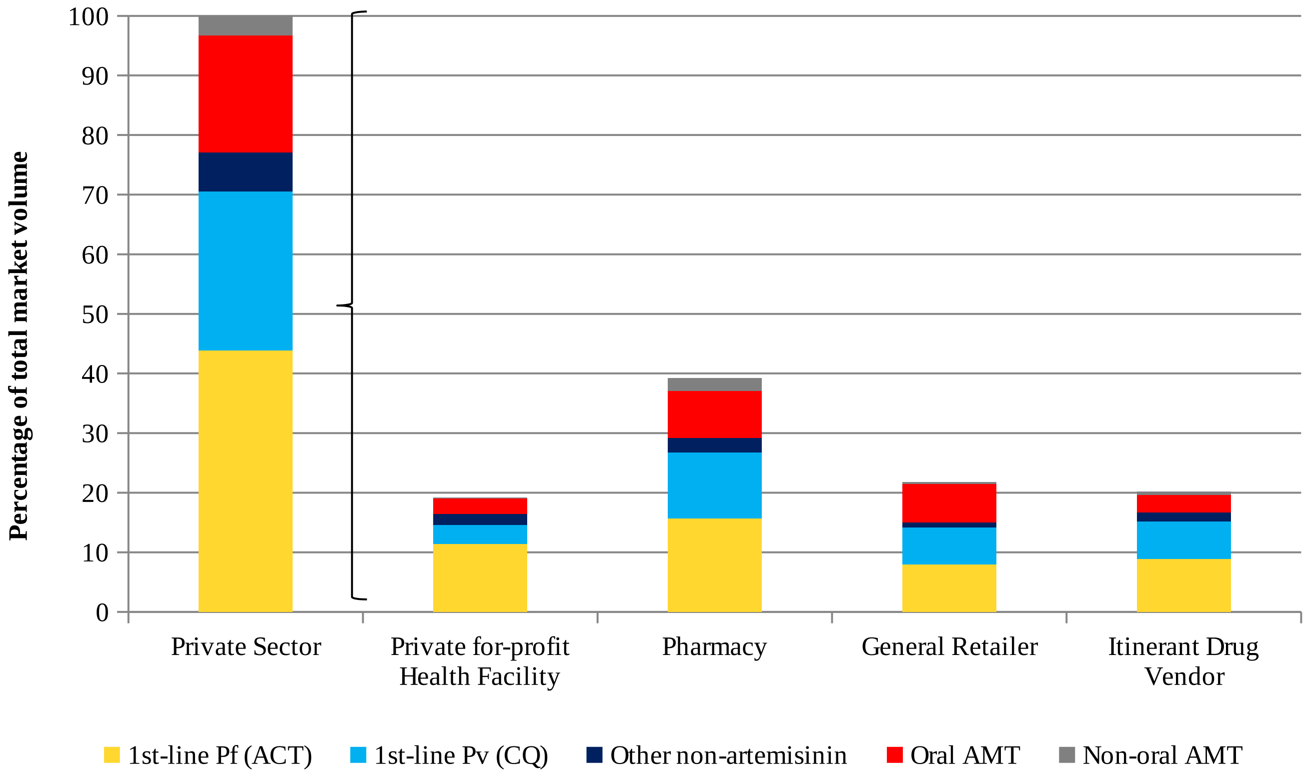

Percentage of total market volume
100
90
80
70
60
50
40
30
20
10
0
Private Sector
Private for-profit Health Facility
Pharmacy
General Retailer
Itinerant Drug Vendor
1st-line Pf (ACT)
1st-line Pv (CQ)
Other non-artemisinin
Oral AMT
Non-oral AMT
